# Late bone metastasis from an apparently benign oncocytic follicular thyroid tumor

**DOI:** 10.1530/EDM-13-0051

**Published:** 2013-09-30

**Authors:** Mauro Boronat, Juan J Cabrera, Carmen Perera, Concepción Isla, Francisco J Nóvoa

**Affiliations:** Section of Endocrinology and NutritionComplejo Hospitalario Universitario Insular Materno-InfantilAvenida Marítima del Sur, s/n. 35016, Las Palmas de Gran CanariaSpain; 1 Service of PathologyComplejo Hospitalario Universitario Insular Materno-InfantilAvenida Marítima del Sur, s/n. 35016, Las Palmas de Gran CanariaSpain; 2 Service of Nuclear MedicineHospital General de Gran Canaria Dr Negrín. Plaza Barranco de la Ballenas/n. 35012, Las Palmas de Gran CanariaSpain; 3 Service of Nuclear MedicineComplejo Hospitalario Universitario Insular Materno-InfantilAvenida Marítima del Sur, s/n. 35016, Las Palmas de Gran CanariaSpain

## Abstract

A man underwent total thyroidectomy for goiter when he was 62 years old. The pathology report informed on a 5.5 cm oncocytic follicular adenoma and a 3.5 mm papillary microcarcinoma. Due to the papillary tumor, he was treated with ablative radioiodine therapy and suppressive doses of levothyroxine. After uneventful follow-up for 9 years, increased levels of serum thyroglobulin were detected. Further imaging studies including a whole body scan (WBS) after an empirical dose of 200 mCi ^131^I were negative. Two years later, a ^99m^Tc SestaMIBI WBS and a 2-[^18^F]-fluoro-2-deoxy-d-glucose positron-emission tomography showed a well-delimited focal uptake in the right femur. A bone biopsy of the lesion demonstrated metastasis of follicular thyroid carcinoma. Retrospective histological reexamination of available material from the primary oncocytic thyroid tumor failed to reveal definitive traits of malignancy.

**Learning points:**

- Oncocytic follicular thyroid tumors are a relatively uncommon variant of follicular thyroid neoplasms mostly composed of distinctive large oxyphilic cells (Hürthle cells).
- Criteria for the distinction between benign and malignant oncocytic neoplasms are not different from those used in the diagnosis of ordinary follicular tumors.
- Some cases of apparently benign oncocytic neoplasms have been found to develop malignant behavior.
- Search to rule out vascular and capsular invasion should be particularly exhaustive in histological assessment of oncocytic thyroid tumors.
- Even so, long-term surveillance remains appropriate for patients with large apparently benign oncocytic tumors.

## Background

Oncocytic thyroid neoplasms are a variant of follicular tumors composed of distinctive large acidophilic cells (Hürthle cells). Just like other types of well-differentiated follicular thyroid tumors, oncocytic neoplasms can be benign (adenomas) or malignant (carcinomas), and the distinction between the two entities is based on identification of capsular or vascular invasion or the presence of metastatic disease. However, difficulty in distinguishing benign from malignant oncocytic follicular tumors has been a matter of long-lasting debate.

## Case presentation

A 73-year-old Italian man was admitted to our Endocrinology Department in April 2010. In May 1999, he had undergone total thyroidectomy for a voluminous goiter incidentally detected on a chest X-ray. According to the pathology report from the attending hospital, the whole left thyroid lobule was occupied by a 5.5×5×4.5 cm oncocytic adenoma. A 3.5 mm incidental papillary microcarcinoma was found in the contralateral lobule. Because of the presence of this papillary microcarcinoma, the patient was treated with an ablative dose of 100 mCi ^131^I. Levothyroxine suppression therapy was instituted from that time onward. According to the institutional protocol, ^131^I whole body scans (WBS) following suspension of levothyroxine were performed in November 1999, October 2000, and July 2003, all of which yielded negative results and undetectable levels of serum thyroglobulin. By August 2008, a ^131^I WBS performed after recombinant human TSH (rhTSH) stimulation also failed to show areas of uptake. However, stimulated serum thyroglobulin was >5000 ng/ml. Cervical ultrasonography and total body contrast computarized tomographic (CT) scan were negative. A 2-[^18^F]-fluoro-2-deoxy-d-glucose positron-emission tomography (FDG-PET) (without rhTSH stimulation) showed increased activity in the left iliac crest. In December 2008, an empirical dose of 200 mCi ^131^I was given after withdrawal of levothyroxine, but post-therapy, WBS was negative. In accordance with the results of FDG-PET, a ^99^Tc bone scintigraphy revealed focally increased uptake of tracer in the left iliac crest, and bone biopsy was undertaken in April 2009, but the histopathological examination was negative for malignancy. Subsequent measures of serum thyroglobulin across the year 2009, while the patient was under suppressive levothyroxine therapy, ranged between 63 and 384 ng/ml. Antithyroglobulin antibodies were invariably negative.

## Investigation

**Figure 1:**
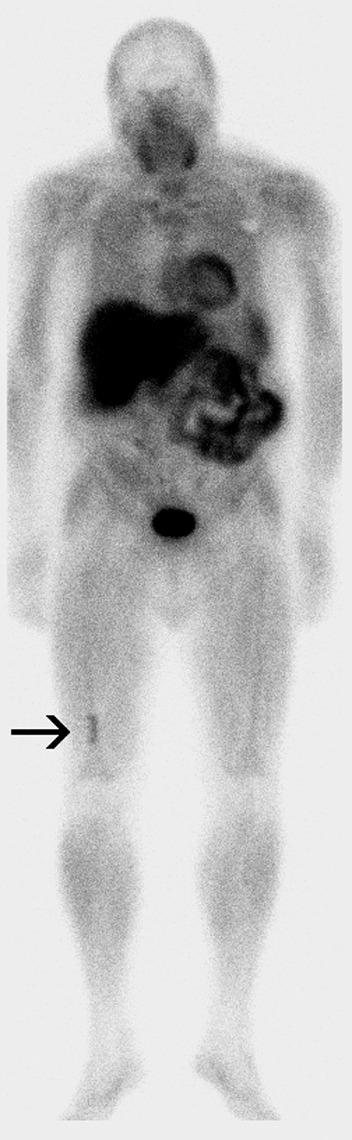
^99^Tc SestaMIBI WBS revealed a focal uptake on the distal third of the right femur.

**Figure 2:**
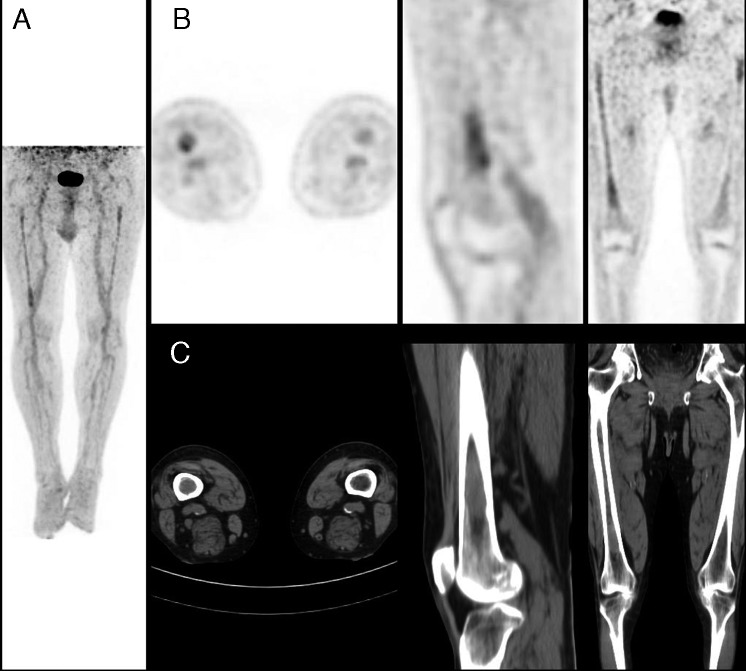
^18^FDG-PET/CT of whole body and lower extremities. (A) Maximum intensity projection image of lower extremities. (B) PET slices: pathological uptake in the distal region of the right femur (SUVmax: 3.6). (C) CT slices: high-density 4.5 cm intramedullary lesion in the right femoral diaphysis.

**Figure 3:**
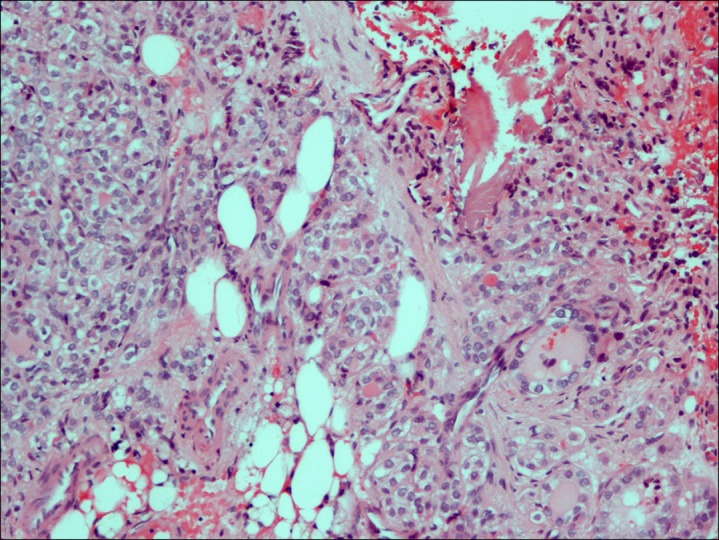
Histology sections of bone biopsy of right femur showing follicular thyroid cells with oncocytic traits among rest of bone marrow tissue and adipose cells (HE×10).

The patient was asymptomatic and physical examination was unremarkable. Serum level of TSH was 0.1 mU/l, free T_4_ 0.91 ng/dl, and thyroglobulin 526 ng/ml. Antithyroglobulin antibodies were undetectable. Cervical ultrasonography and thoraco-abdominal CT scan were negative. A ^99m^Tc SestaMIBI WBS disclosed a well-delimited focal uptake in the distal region of the right femur (Fig. 1). A FDG-PET/CT study performed after rhTSH stimulation showed lytic resorption in the left iliac crest, just above the supra-acetabular margin, but there was no definitely increased FDG uptake in this area on the PET image. However, PET images showed intense ^18^F-FDG uptake in the distal intramedullary region of the right femur (SUVmax=3.6), corresponding to a high-density lesion measuring 4.5 cm of craniocaudal diameter in the CT study (Fig. 2). CT-guided bone biopsy of the femoral lesion demonstrated metastasis of follicular thyroid carcinoma (Fig. 3). Immunohistochemical studies showed no staining for cytokeratin 19, a characteristic marker of papillary carcinoma, and a final diagnosis of oncocytic thyroid carcinoma with distant bone metastasis was established.

**Figure 4:**
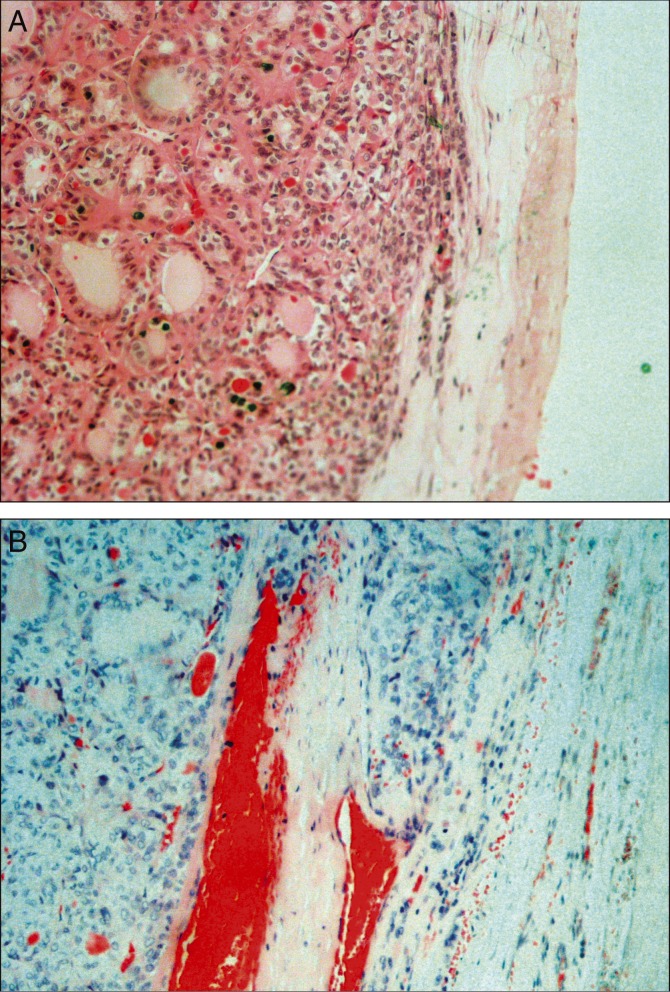
Histology section of the primary tumor. (A) The nodule was composed of areas with solid or microfollicular architecture and was circumscribed by a well-delimited fibrous capsule (HE×10). (B) Definite capsular or vascular invasion was not observed, although vascular compression and incomplete capsular invasion were present in certain areas of the lesion (HE×10).

Histological material from the primary tumor was requested and kindly submitted from the first attending hospital. Microscopic examination of submitted slides showed an oncocytic follicular tumor surrounded by a fibrous capsule (Fig. 4A). For the most part, tumoral cells were arranged in a dense solid pattern of growth. Protrusion on the inner aspect of the capsule was present in some areas of the tumor, along with compression to adjacent vessels (Fig. 4B). However, neither capsular invasion nor vascular permeation by tumor cells was definitely recognized and it was not possible to demonstrate unequivocal signs of invasiveness. Additionally, a 0.35×0.3 cm focus of papillary microcarcinoma, composed of papillary formations and follicular elements with cells containing typical clear nuclei, was also detected.

## Treatment

The patient was treated with external beam radiation therapy (8 Gy) to the right femur and monthly administration of i.v. zoledronic acid.

## Outcome and follow-up

Three other PET/CT studies were done 6, 12, and 24 months later. Imaging of the bone metastasis was stable and no new lesions have appeared. As the patient continues doing well, no additional therapeutic measures have been undertaken up to now.

## Discussion

Historically, there has been controversy over the accuracy of histological criteria to differentiate between benign and malignant oncocytic follicular thyroid tumors. Debate was fired up in the 1970s with the publication of some cases of oncocytic neoplasms considered benign by histological criteria that subsequently developed malignant behavior, leading to metastatic disease and death of the patients (1). The authors suggested that the term ‘Hürthle cell adenoma’ should be abandoned. They claimed that all Hürthle cell tumors were potentially malignant and advocated total thyroidectomy for all such lesions. This report prompted the publication of a considerable number of case series analyzing the outcome of surgically treated oncocytic neoplasms. Although very occasional cases of histologically benign lesions were subsequently proven to have a Hürthle cell follicular carcinoma (2) (3) (4), reported data demonstrated that strict histological criteria (presence or absence of capsular and/or vascular invasion) should be able to differentiate Hürthle cell carcinoma from adenoma in virtually all cases. For the time being, it is definitely accepted that criteria used for diagnosis of the oncocytic variant of follicular thyroid carcinoma should not be different from those used in the diagnosis of ordinary follicular carcinomas (5). Nevertheless, while differential diagnosis between follicular adenomas and minimally invasive encapsulated carcinomas represents a challenging task for any type of follicular tumors, the most reputed pathologists still recognize that signs of invasiveness are more frequent among oncocytic tumors than in conventional follicular tumors and recommend an even more exhaustive search of vascular and capsular invasion in these cases (6).

The present case rekindles concerns about the possibility of misdiagnosing an oncocytic follicular carcinoma as a benign lesion. Our patient underwent total thyroidectomy for removal of an oncocytic tumor that was deemed benign in his first attending hospital. Striking elevation of serum levels of thyroglobulin, suggesting hidden metastatic disease, was detected 9 years later, and definite confirmation of bone metastasis was performed after another 2 years. In spite of this clinical evidence, a second retrospective through revision of histological slides from the primary tumor in our center failed to find definite traits of malignancy. Obviously, it is possible that areas of microinvasion could be present in other sites not represented on the examined histological sections. It has been previously shown that, when examining borderline follicular neoplasms, the more sections assessed, the higher chance of finding carcinoma (7). Alternatively, if assumed that unequivocal capsular/vascular invasion could not be definitely demonstrated, a diagnosis of ‘follicular tumor of uncertain malignant potential’ could have been considered, although this term was not in use when the patient underwent thyroidectomy (5). Nonetheless, for any of these two assumptions (i.e., a minimally invasive carcinoma without angioinvasion or a tumor of uncertain malignant potential), the clinical behavior as observed in the present case would be unexpected, as the recurrence rate of these types of neoplasms is close to zero (8).

It must be noted that, although they *per se* cannot be directly considered a criterion for diagnosis of carcinoma, several clinical and pathological features of this case have been shown to be associated with greater risk of malignancy and should raise a high index of suspicion. Specifically, older age and tumor size have been associated with an increased probability of malignancy among subjects harboring oncocytic neoplasms (9). In addition, the presence of a solid growth pattern, such as the one described in the present report, was found to increase the risk of recurrence in a specific series of encapsulated minimally invasive follicular oncocytic tumors (8).

Other several clinical aspects of this case also deserve consideration. First, although the primary tumor was misdiagnosed as a benign lesion, the patient was treated with total thyroidectomy and postsurgical ablation with radioiodine because of the presence of an incidental papillary microcarcinoma. Neither guidelines from the American Thyroid Association nor the European Thyroid Association consensus advocate the use of radioiodine for papillary microcarcinomas. In fact, this could be considered an aggressive treatment even for a minimally invasive follicular tumor. Moreover, a second empirical dose of ^131^I was administered 9 years later, when elevation of serum thyroglobulin was detected. Nevertheless, in spite of these therapeutic procedures, the outcome was unfavorable. No radioiodine uptake was visible in post-therapeutic WBS and levels of thyroglobulin did not lower after the administration of ^131^I. This is not surprising, as ability to take up radioiodine is unusual for oncocytic carcinomas. Our case illustrates that ^99m^Tc Sestamibi WBS and FDG-PET can be useful to localize metastases from oncocytic tumors when ^131^I scan is negative (10).

In summary, distinguishing benign from malignant oncocytic follicular neoplasms remains difficult. It seems still prudent to recommend a very long period of surveillance for large solid non-malignant-appearing oncocytic tumors.

## Patient consent

Written informed consent has been obtained from the patient for publication of this article and accompanying images.

## Author contribution statement

M Boronat is the patient's physician and wrote the manuscript. J J Cabrera performed the pathological studies. C Perera and C Isla performed the nuclear imaging studies. F J Nóvoa gave clinical advice, reviewed the manuscript, and contributed to the discussion.
